# Efficacy and safety of apatinib in the treatment of osteosarcoma: a single-arm meta-analysis among Chinese patients

**DOI:** 10.1186/s12885-021-08154-3

**Published:** 2021-04-23

**Authors:** Hui Yao, Xuyu Chen, Xiaodong Tan

**Affiliations:** School of Health Sciences, Wuhan University, Wuhan, China

**Keywords:** Apatinib, Osteosarcoma, Meta-analysis

## Abstract

**Background:**

Osteosarcoma is a relatively rare malignant tumor with a high incidence in young people. The development of tyrosine kinase inhibitors has brought the treatment of osteosarcoma into a new stage. Apatinib, a tyrosine kinase inhibitor specifically targeting VEGFR2, has been increasingly reported as a treatment for osteosarcoma with promising outcome parameters, but there has been no systematic analysis of the treatment of osteosarcoma by apatinib.

**Methods:**

A single-arm meta-analysis was performed, and published literature from PubMed, Web of Science, Embase, Cochrane Library, CNKI and Wan Fang databases as of March 1, 2021 was systematically retrieved. Quality assessment is carried out in accordance with a 20 item checklist form prepared by the Institute of Health Economics (IHE). Double arcsine transformation is performed to stabilize the variance of the original ratio. When I^2^ > 50%, the random effect model is used to calculate the pooled parameters; otherwise, the fixed effect model is used. We conducted subgroup analysis according to age and apatinib dose.

**Results:**

This meta-analysis included 11 studies of 356 Chinese patients with osteosarcoma. The pooled objective remission rate (ORR) of advanced or metastatic osteosarcoma treated by oral apatinib in Chinese patients was 0.27(95%CI = 0.18–0.38). The pooled disease control rate (DCR) was 0.57 (95%CI = 0.42–0.72). The pooled median progression-free survival (mPFS) and median total survival (mOS) were 5.18 months (95%CI = 4.03–6.33) and 10.87 months (95% CI = 9.40–12.33), respectively. More than 70% of adverse reactions were mild, the most common adverse reaction was hand-foot syndrome (HFMD), with an incidence of 0.46 (95%CI = 0.35–0.58), the second was hypertension, with an incidence of 0.40 (95%CI = 0.29–0.51).

**Conclusions:**

The efficacy of apatinib in the treatment of osteosarcoma is competitive with current evidence, and it is worth noting that its low cost can significantly improve patient compliance and increase therapeutic value.

## Introduction

Osteosarcoma is a relatively rare malignancy that occurs mostly in children and adolescents, with an incidence of about 3 per million persons every year in China [1]. As one of the most common bone malignant tumors, it originates from the stromal tissues and the most frequently found site was long shaft scale, especially around the knee joint [2]. Although the combination of resection and adjuvant and neoadjuvant chemotherapy has made great progress since the 1970s, the 5-year survival rate has increased from less than 20% to 60%–80%, the survival rate for metastatic or relapse osteosarcoma is still not optimistic [3–8]. The development of molecular targeted drugs in malignant tumors has brought the treatment of osteosarcoma to a new stage [9–11]. Previous studies have shown that tyrosine kinase inhibitors such as geffitinib, sorafenib and apatinib show promising potential in inhibiting metastasis and invasion of osteosarcoma [12–14]. Apatinib, a small molecule targeted anti-angiogenesis drug, was approved in China in 2014 for the treatment of advanced gastric cancer and has shown good safety and efficacy [15]. The oral preparation of apatinib and its low price can effectively improve patient compliance and reduce treatment cost. In addition to its extensive application in the treatment of gastric cancer, apatinib has made positive progress in clinical trials for a variety of cancers, including osteosarcoma and soft tissue sarcoma [16–20].

The expression of vascular endothelial growth factor receptor 2 in osteosarcoma tissues was significantly higher than that in normal bone tissues, and the patients with high expression level had a poor prognosis [21]. Apatinib strongly inhibits tumor angiogenesis by highly selective competition of ATP binding sites for VEGFR-2 in cells [22]. In addition, apatinib has been shown to inhibit osteosarcoma cell proliferation and induce osteosarcoma cell apoptosis and G0/G1 phase arrest in vitro, and inhibit cell invasion, migration and PD-1 expression, and the STAT3/ Bcl-2 signaling pathway is considered as a possible mechanism [21, 23].

Although some studies have shown that apatinib is effective in the treatment of osteosarcoma, especially advanced osteosarcoma, it must be said that the current mechanism of exploration and clinical trials are limited, and need to be further enriched. To our knowledge, there have been no systematic analysis reports of apatinib in the treatment of osteosarcoma, This meta-analysis examines the efficacy and safety of apatinib in the treatment of osteosarcoma patients in China, providing a reference for clinicians to make the best choice in clinical practice.

## Methods

### Search strategy

A systematical search to retrieve published literatures from PubMed, Web of Science, Embase, Cochrane Library, China National Knowledge Infrastructure (CNKI) and Wan Fang databases was conducted up to March 1, 2021. There was no language restriction in this meta-analysis. The following keywords were used: “Osteosarcoma [MeSH Terms]”, “Osteosarcoma Tumor”, “Osteosarcoma Tumors”, “Tumor, Osteosarcoma”, “Tumors, Osteosarcoma”, “Sarcoma, Osteogenic”, “Osteogenic Sarcomas”, “Sarcomas, Osteogenic”, “Osteogenic Sarcoma”, “apatinib”, “apatinib mesylate” and “YN968D1”.

### Study selection

The following inclusion criteria were used in this study: (1) All study types except case reports were considered for inclusion (e.g. prospective or retrospective cross-sectional cohort studies and case-control studies). (2) The study participants were Chinese patients with osteosarcoma. (3) The study clearly reported the age of the participants and the dosage of the treatment medication. Duplicate studies and studies that contain other tumors and whose data cannot be extracted separately are excluded.

Two authors (HY and XC) screened the titles and abstracts of all retrieved studies based on search strategies, and eliminated the studies that obviously did not meet the inclusion criteria. Information and data were extracted by two authors (HY and XC) from studies that met the inclusion criteria independently. Any disagreements were resolved by discussion with a third investigator (XT). The characteristics of the included studies are summarized as follows: first author name, year of publication, study type, number of cases, patient age, apatinib dosages, outcome parameters.

### Outcome definitions

Clinical responses included objective response rate (ORR), disease control rate (DCR), median progression-free survival (mPFS), median overall survival (mOS), and adverse reactions. All adverse reactions are classified into grades 1–2 and 3–4, including fatigue, pain, hypertension, hand-foot syndrome, rash, diarrhea, anorexia, weight loss, pneumothorax, wound healing problems, oral mucositis, proteinuria, etc.

### Quality assessment

A relatively systematic and comprehensive quality assessment tool developed by the Canadian Institute of Health Economics (IHE) for the case series was applied in quality assessment [24]. Considering that it may be misleading to score the conformity of items, the list gives corresponding options for each item to enhance the objectivity of scoring, and studies that met 14 or more of the 20 items (70% or more) were considered to be of acceptable quality.

### Statistical analysis

For the original data that do not conform to the normal distribution, double arcsine transformation is performed to stabilize the variance of the original ratio. Heterogeneity assessment included chi-square test and I^2^ value. *P* < 0.1 indicated a statistically significant difference. When I^2^ is greater than 50%, the combined proportion and 95% confidence interval are calculated by the random effects model. Otherwise, a fixed effect model is used. Considering the limited statistical efficiency of the chi-square test and the limited number of studies included in our research, *P* value of 0.10 was adopted as the significance level rather than the conventional level of 0.05 to increase the test efficiency. Potential sources of heterogeneity were investigated by subgroup.

## Results

### Search results

**Fig. 1:**
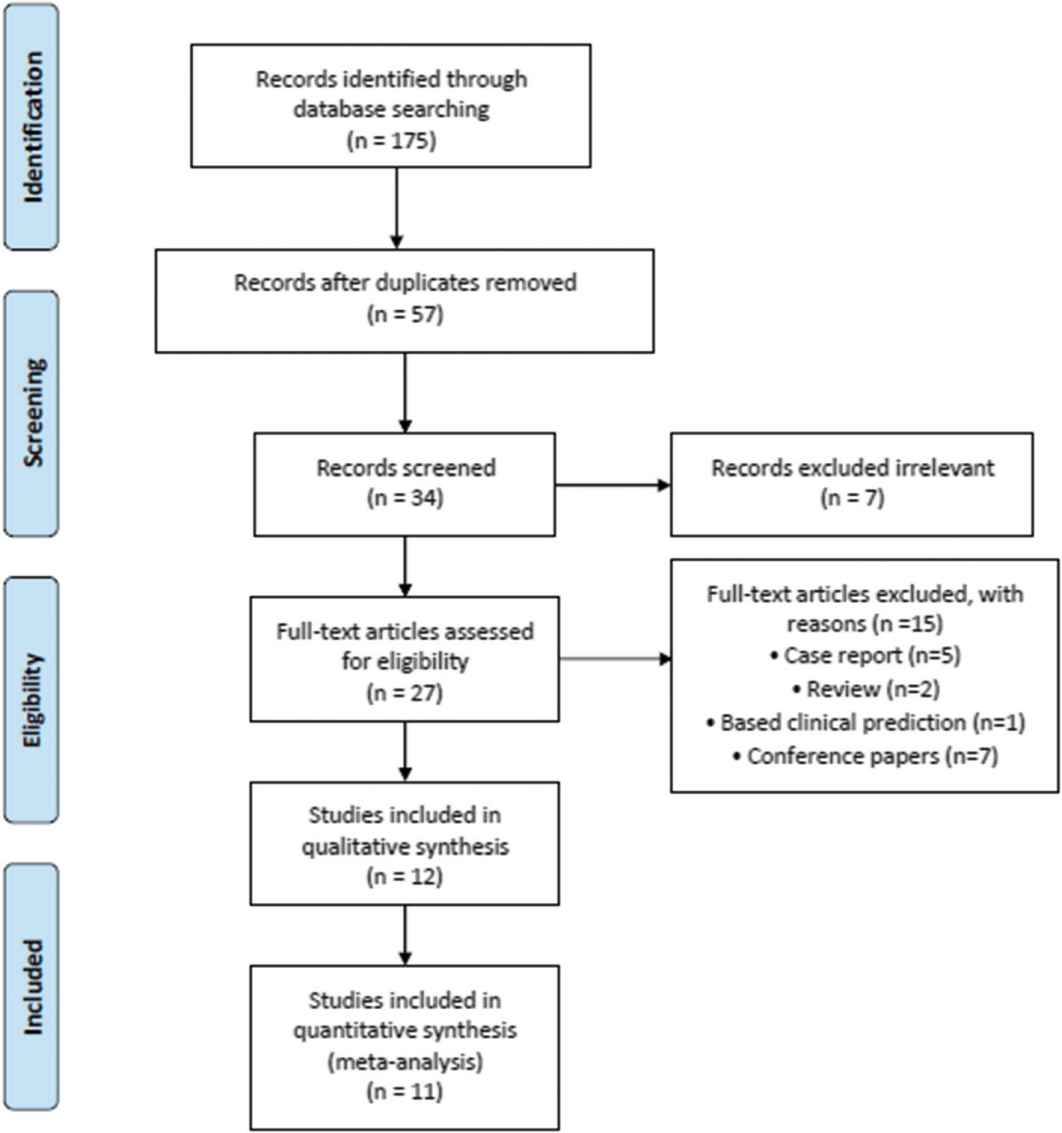
Flow diagram of the selection process

Our initial search found a total of 175 studies. After excluding repetitive studies, 57 were left. After screening the titles and abstracts, we excluded 7 irrelevant studies. 5 case reports, 2 reviews, 1 clinical prediction model study and 7 conference papers were eliminated by reading the full text (Fig. 1). Finally, a total of 11 studies involving 356 Chinese patients with osteosarcoma were eventually included in this meta-analysis [25–35].

### Patient characteristics and quality assessment

All 11 eligible studies were conducted in China, including 9 retrospective studies and 2 phase II clinical trials. All patients had advanced osteosarcoma or metastasis and were treated with oral apatinib at doses ranging from 250 mg to 750 mg. As shown in Table 1, the quality assessment scores of all included studies were 14 or above. Details of all studies and the characteristics of the patients with osteosarcoma are shown in Table 2.

**Table 1:**
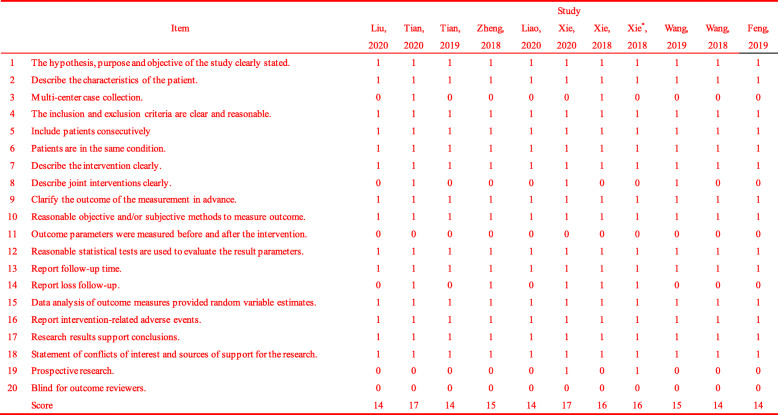
Quality assessment results of included studies by IHE case series quality assessment tool

**Table 2:**
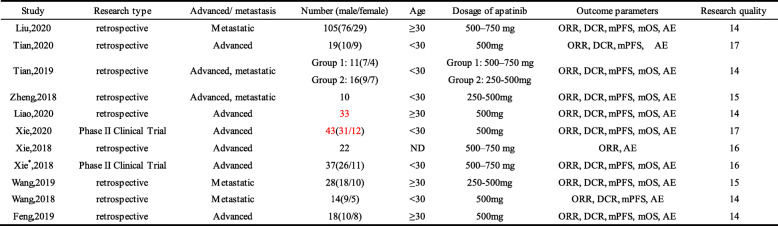
Clinical information and patient characteristics from the eligible studies

Tian,2019: The study was divided into two groups based on the dose of apatinib, with group 1 as the high-dose group and group 2 as the low-dose group

### Therapeutic efficacy assessments

#### ORR

**Fig. 2:**
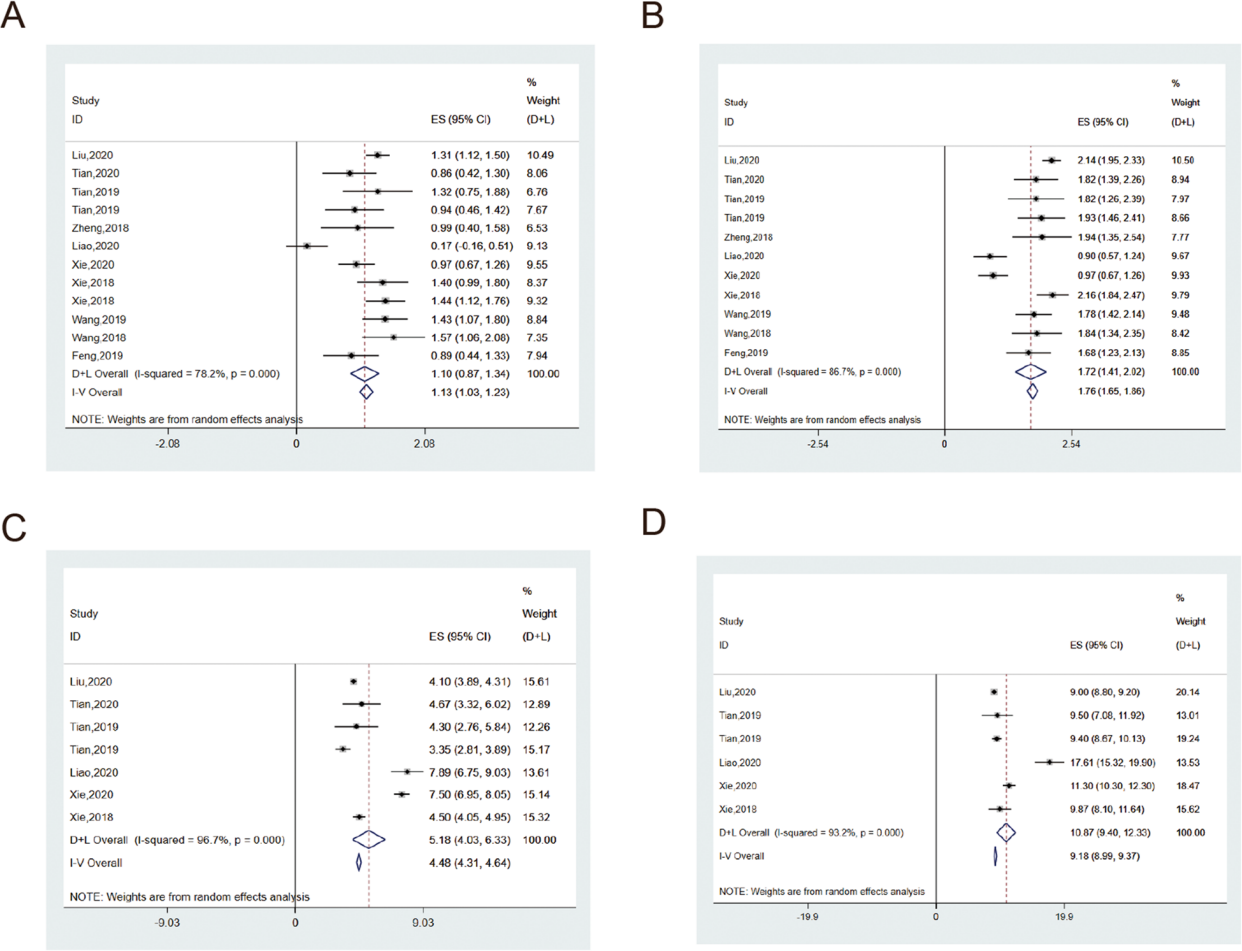
The ORR (**a**), DCR (**b**), mPFS(**c**) and mOS(**d**) of apatinib in the treatment of osteosarcoma

The pooled objective remission rate (ORR) of advanced or metastatic osteosarcoma treated by oral apatinib in Chinese patients was 0.27 (95%CI = 0.18–0.38), with high inter-study heterogeneity (I^2^ = 78.2%, *p* = 0.00) (Fig. 2a). After the exclusion of a study in which the effective number of objective responses was 0, the pooled ORR was recalculated to 0.33(95%CI = 0.28–0.38), indicating a significant decrease in heterogeneity (I^2^ = 38.1%, *p* = 0.10). The pooled ORR of age group above 30 (0.21, 95%CI = 0.04–0.47) was lower than that of the age group below 30 (0.29, 95%CI = 0.20–0.40). Based on the dose subgroup, ORR of 500-750 mg group was the highest (0.39, 95%CI = 0.32,0.46) and the heterogeneity level was low (I^2^ = 0.0%, *p* = 0.92), ORR of 500 mg group was the lowest (0.17, 95%CI = 0.04–0.37) and the heterogeneity was still high (I^2^ = 83.1%, *p* = 0.00), ORR of 250-500 mg group was 0.30(95%CI = 0.16–0.47). The results were similar to those above after excluding a study with 0 objective responders, which had a greater influence on heterogeneity. (Tables 3, 4, and 5).

**Table 3:**
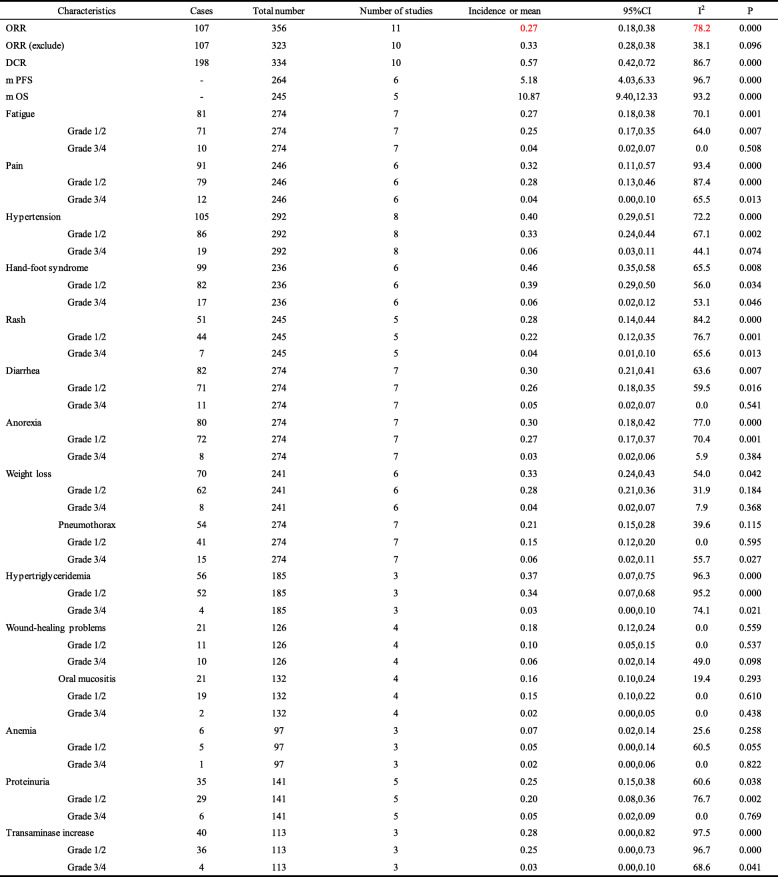
ORR, DCR, mPFS, mOS and adverse reactions of apatinib in the treatment of osteosarcoma

ORR (exclude): One study with effective value is 0 is excluded

**Table 4:**
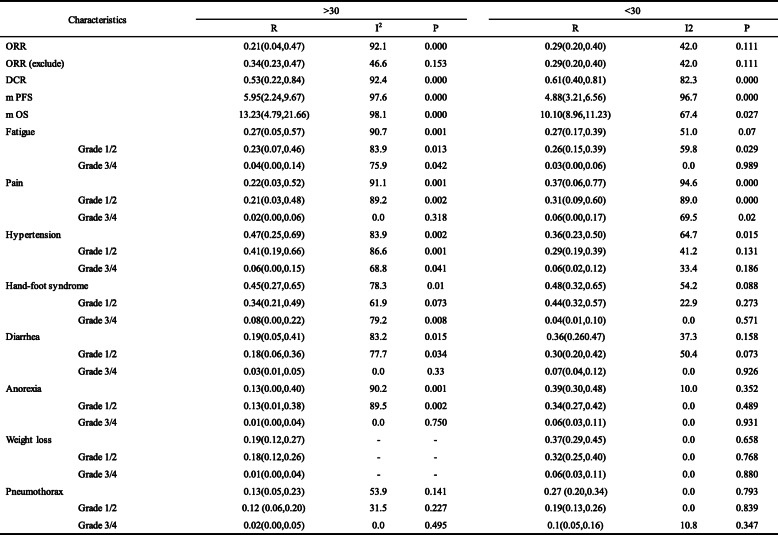
Age subgroup analysis of ORR, DCR, mPFS, mOS and adverse reactions

ORR (exclude): One study with effective value is 0 is excluded

Subgroup analysis was performed when the number of included studies was greater than 5

**Table 5:**
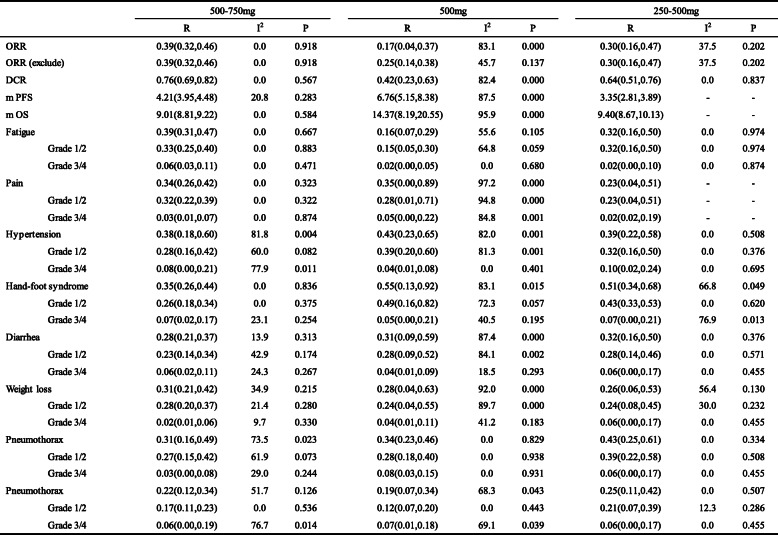
Dose subgroup analysis of ORR, DCR, mPFS, mOS and adverse reactions

ORR (exclude): One study with effective value is 0 is excluded

Subgroup analysis was performed when the number of included studies was greater than 5

#### DCR

A total of 10 studies included DCR parameters, with a pooled value of 0.57 (95%CI = 0.42–0.72) (Fig. 2b). DCR of age groups were similar to ORR, the age group above 30 years old (0.53, 95%CI = 0.22–0.84) was lower than that under 30 years old (0.61, 95%CI = 0.40–0.81), with high level of intergroup heterogeneity (92.4% and 82.3%). DCR was highest in the 500-750 mg dose group (0.76, 95%CI = 0.69–0.82), followed by the 250-500 mg dose group (0.64, 95%CI = 0.51–0.76) and the 500 mg group (0.42, 95%CI = 0.23–0.63). The 500 mg group explained 95% of the sources of heterogeneity (I^2^ = 82.4%, *p* = 0.00). (Tables 2, 3, and 4).

#### mPFS

The pooled median progression-free survival (mPFS) of the 6 included studies was 5.18 months (95CI = 4.03–6.33), with a high level of inter-study heterogeneity (I^2^ = 96.7%, *p* = 0.00) (Fig. 2c). The pooled mPFS was 5.95 (95%CI = 2.24–9.67) for the age group over 30 and 4.88 (95%CI = 3.21–6.56) for the age group under 30. The 500 mg group showed the highest pooled mPFS of 6.76 months (95%CI = 5.15–8.38), followed by the 500-750 mg group for 4.21 months (95%CI = 3.95–4.48) and finally the 250-500 mg group for 3.35 months (95%CI = 2.81–3.89). After dose subgroup analysis, the heterogeneity was significantly higher in the 500 mg group. (Tables 3, 4, and 5).

#### mOS

The pooled median overall survival (mOS) was 10.87 months (95% CI = 9.40–12.33) and a total of 5 studies were included with inter-study heterogeneity of 93.2% (Fig. 2d). In the age group older than 30 years, mOS was 13.23 months (95%CI = 4.79–21.66), OS was 10.10 months (95%CI = 8.96–11.23) in the age group less than 30 years old, and the heterogeneity between the two groups was relatively high. mOS in the 500 mg–750 mg group, 500 mg group, and 250-500 mg group were 9.01, 14.37, and 9.4 months, respectively, and the source of heterogeneity was mainly in the 500 mg group. (Tables 3, 4, and 5).

### Adverse events

The present study also assessed the incidence of adverse events in patients with advanced osteosarcoma treated with apatinib, with all adverse events classified as grade 1/2 or grade 3/4, more than 70% of the adverse reactions were grade 1/2. Common adverse reactions include hand-foot syndrome, high blood pressure, pain, fatigue, etc. The most common adverse reaction was hand-foot syndrome (HFMD), with an incidence of 0.46 (95%CI = 0.35,0.58), the second was hypertension, with an incidence of 0.40 (95%CI = 0.29–0.51). The incidence of hypertriglyceridemia, weight loss, pain, anorexia, and diarrhea was high at 0.37 (95%CI = 0.07–0.75), 0.33 (95%CI = 0.24–0.43), 0.32 (95%CI = 0.11–0.57), 0.30 (95%CI =0.18–0.42), and 0.30 (95%CI =0.21–0.41), respectively. The incidence of fatigue, rash, pneumothorax, and proteinuria was 0.27 (95%CI = 0.18–0.38), 0.28 (95%CI = 0.14–0.44), 0.21 (95%CI = 0.15–0.28), and 0.25 (95%CI = 0.15–0.38), respectively. The study also reported some unusual adverse events (less than 50% of the original studies reported), with rates of wound healing problems, oral mucosal inflammation, elevated transaminases, and anemia of 0.18 (95%CI = 0.12–0.24), 0.16 (95%CI = 0.10–0.24), 0.28 (95%CI = 0.00–0.82), and 0.07 (95%CI = 0.02–0.14), respectively. (Tables 3, 4, and 5).

### Subgroup analysis

A subgroup analysis was performed to explore the sources of heterogeneity in patient age and apatinib dose. The heterogeneity of the group under 30 years old was significantly reduced (I^2^ = 42.0, *p* < 0.111). Group under 30 years old showed higher ORR and DCR, but the performance of mPFS and mOS were not as good as group over 30 years old and the incidence of adverse reactions such as rash, diarrhea and anorexia was higher. Dose subgroup analysis showed that the heterogeneity of ORR, DCR, mPFS, and mOS in the high-dose group was significantly reduced. The ORR and DCR of the high dose group (500 mg–750 mg group) were optimal, mPFS and mOS of the medium-dose group (500 mg) were optimal while the ORR and DCR parameters performed the worst. Results of the subgroup analysis are depicted in Table 4 and 5.

## Discussion

Osteosarcoma are generally locally aggressive, and 10–20% of patients who are diagnosed for the first time have had early metastases, and in severe cases potentially fatal systemic metastases, mainly to the lungs and bones [36–38]. The incidence of lung metastasis reported in this study was as high as 90–100%, and the significantly higher incidence level may be related to the fact that all the patients in this study were patients with advanced or metastatic osteosarcoma. It has been reported that high expression of IMP3 and VEGF is associated with an increased possibility of lung metastasis, significantly shortened survival time, and stage of osteosarcoma [39].

Cabozantinib is a tyrosine kinase inhibitor similar to apatinib. A recent phase clinical trial reported the clinical effect of cabozantinib in the treatment of 42 patients with advanced or metastatic osteosarcoma. The objective response rate was 0.12, which was significantly lower than the objective response rate of apatinib in the treatment of osteosarcoma, with a pooled mean of 0.28. Median progression-free survival and total survival were 6.7 months (5.4–7.9) and 10.6 months (7.4–12.5), which were superior to the 5.18 months and slightly lower than the 10.87 months in the meta-analysis, respectively [40]. This may be related to the number of targets for both drugs, with apatinib identifying fewer targets than other tyrosine kinase inhibitors. Apatinib inhibits the activity of vascular endothelial growth factor receptor-2 tyrosine kinase highly selectively, and blocks the signal transduction of the binding of vascular endothelial growth factor to its receptor, thereby inhibiting the angiogenesis of osteosarcoma and exerting anti-tumor effects. However, carbotinib has fairly multiple anti-tumor targets, including VEGFR2, MET, PDGFRβ, and so on [20, 41].

The longer median progression-free survival suggests that capotinib currently offers the best treatment for osteosarcoma, although the trial is a multi-center collaboration, considering that the number of patients participating in the study is limited and there are no similar reports, further clinical effects remain to be verified. The overall incidence of adverse reactions of apatinib in the treatment of osteosarcoma is lower than that of cabotinib, both of them have a high incidence of diarrhea. In addition, patients treated with capatinib appeared to be more prone to oral mucositis, with an incidence of up to 47%, however, treatment with apatinib has been infrequently reported, with an incidence of only 16%.

We also conducted subgroup analysis and exploration of sources of heterogeneity. We found the exclusion of a study with an objective response rate of 0 led to a significant decrease in heterogeneity, and subsequent subgroup analysis also supports this result. Although the heterogeneity of excluding 0 event decreased, it is obviously not desirable, other studies have given the same suggestions [42, 43]. Subgroup analysis of age showed that the heterogeneity of the objective response rate was significantly reduced, and objective response rate and disease control rate were higher in younger patients, which is consistent with the findings of Jain et al. [44]. Subgroup analysis of drug dosage showed that the heterogeneity of ORR, DCR, mPFS, and mOS was significantly reduced, especially in the high-dose group. The degree of heterogeneity decrease in the medium-dose group is limited, which may be caused by the same dose of all patients in the medium-dose group without considering the patient’s baseline physical condition (such as body surface area) to determine the dose. Thus, patient age and medication dosage are potential sources of heterogeneity.

Our meta-analysis showed that objective response rates and disease control rates were better in patients under 30 years of age, but median progression-free survival and median overall survival were better in patients over 30 years of age. The meta-analysis based on dose groupings showed that the objective response rate and disease control rate were optimal in the high-dose group, and the median progression-free survival and median overall survival were optimal in the middle-dose group. It appears that the objective response rate and median progression-free survival are not optimal at the same time, similar study has shown that this may not only in different age groups and dose groups, but also in different treatment regimens [40].

Drug safety is the top priority in treatment. The results of this meta-analysis showed that the low incidence of adverse reactions, mainly mild adverse reactions, and tolerable and controllable adverse reactions showed great advantages in the treatment of osteosarcoma with apatinib. This is consistent with previous studies that the highest incidence is hand-foot syndrome and hypertension [45]. A study of regofenil for osteosarcoma reported adverse events that were similar to this study, including fatigue, diarrhea, and weight loss, but at higher levels than apatinib. In particular, the incidence of fatigue and diarrhea of Regolfini was as high as 89% and 45%, compared with 27% and 30% of apatinib, based on the results of this study [9].

Based on the great advantages of apatinib in the treatment of osteosarcoma, researchers began to explore the efficacy of apatinib-encapsulated hydrophobic poly nanoparticles in the treatment of advanced or refractory osteosarcoma. This regimen has been demonstrated to have the potential to improve the efficiency of targeted therapy and reduce toxicity [46]. Other researchers have explored the use of apatinib in combination with other drugs to treat osteosarcoma. The combination seemed to prolong progression-free survival, but the effect was limited, with 60% of patients failing to achieve progression-free survival at 6 months [33]. Therefore, the path to improve the efficiency of apatinib in the treatment of osteosarcoma needs to be further explored.

Our study has several limitations. First, the purpose of this study was to investigate the efficacy of apatinib in patients with advanced osteosarcoma. However, for some reasons (such as ethical issues), there have been few randomized controlled trials focusing on the effect of apatinib on important outcomes in patients with advanced osteosarcoma. Case series may be the only available evidence, so most of the studies included in this meta-analysis were observational studies without a control group. As more and more clinical small sample studies need meta-analysis to obtain high-quality evidence, corresponding analysis methods need to be developed. Considering that there is still a lack of evidence from randomized controlled trials, we will follow up relevant evidence reports in the future, and hope that this study can provide a reference for subsequent clinical trials. Second, there were 2 studies with drug combination which may bias the results. Study of Wang et al., patients received apatinib daily plus chemotherapy. The chemotherapy regimen is 21 days as a cycle with 75 mg·m^− 2^ docetaxel at day 1 and 30 mg·m^− 2^ cisplatin at day 1–4. In the study of Xie et al. patients received apatinib orally once daily plus camrelizumab by intravenous infusion every 2 weeks until disease progression or unacceptable toxicity. Based on the treatment regimen, it should be understood that apatinib is the main drug and camrelizumab is the adjuvant drug, similarly, chemotherapy is auxiliary. Considering the patient type and disease progression are consistent, the outcome parameter report is complete and the insufficient amount of available evidence currently, we retain these two studies after comprehensive consideration and further studies can be carried out after more and more evidence reports of combination medications.

## Conclusion

Apatinib is a targeted drug widely used in tumor treatment. It has been successfully applied to the treatment of osteosarcoma and has shown great advantages. This study pioneered a systematic review of the efficacy and safety of apatinib in the treatment of osteosarcoma. This provides a new reference for the clinical treatment of patients with osteosarcoma, especially patients with advanced and metastatic osteosarcoma.

Compared with current evidence of optimal treatment, apatinib for osteosarcoma presents a competitive therapeutic efficacy with a good median progression-free survival and median overall survival, excellent objective remission rates, lower incidence and severity of adverse events. Patient age and medication dosage are potential factors for the effect. It is also worth pointing out that considering its low price, it can greatly improve patient compliance and increase the value of treatment.

## Data Availability

Not applicable.
